# Chondroitin Sulfates Control Invasiveness of the Basal-Like Breast Cancer Cell Line MDA-MB-231 Through ROR1

**DOI:** 10.3389/fonc.2022.914838

**Published:** 2022-05-31

**Authors:** Satomi Nadanaka, Jun-ichi Tamura, Hiroshi Kitagawa

**Affiliations:** ^1^ Laboratory of Biochemistry, Kobe Pharmaceutical University, Kobe, Japan; ^2^ Department of Agricultural, Life and Environmental Sciences, Faculty of Agriculture, Tottori University, Tottori, Japan

**Keywords:** proteoglycan, chondroitin sulfate, breast cancer, receptor tyrosine kinase-like orphan receptor 1, Dickkopf 1

## Abstract

Extracellular and cell surface chondroitin sulfates (CSs) regulate cancer cell properties, including proliferation and invasion. Thus, it is necessary to understand the mechanisms underlying their roles in cancer. Although we have shown that CS has an inherent ability to enhance the invasive activity of the human triple-negative breast cancer cell line MDA-MB-231, its molecular mechanism remains elusive. Here, we focused on receptor tyrosine kinase-like orphan receptor 1 (ROR1) and dickkopf WNT signaling pathway inhibitor 1 (DKK1). MDA-MB-231 cells express high levels of ROR1; their invasive potential depends on ROR1 signaling. Although accumulating evidence has demonstrated that ROR1 is associated with aggressive breast-cancer phenotypes, the whole picture of its biological function remains poorly understood. In this study, we examined whether CS controls ROR1 function. Surface plasmon resonance analysis indicated that CSs were bound to ROR1 in the presence of WNT5A. The invasive activity of MDA-MB-231 cells enhanced by CSs was completely suppressed by *ROR1* knockdown. In addition, knockdown of the CS biosynthetic enzymes CHST11 and CHST15 inhibited invasive activity, even in the presence of ROR1. These results suggest that CS is required to induce an ROR1-dependent, aggressive MDA-MB-231 phenotype. ROR1 signaling in MDA-MB-231 cells activated c-Jun N-terminal kinase (JNK), leading to increased invasive potential; moreover, exogenous CSs activated JNK. MDA-MB-231 cells express DKK1, a tumor suppressor factor that binds to CS, at high levels. Knockdown of *DKK1* enhanced CS-stimulated tumor invasion activity of MDA-MB-231 cells, suggesting that DKK1 sequesters CS to block ROR1/JNK signaling. These results showed that CSs promotes cancer aggressiveness through the ROR1−JNK axis in MDA-MB-231 cells.

## Introduction

Tumor-associated glycocalyx plays a key role in the promotion and regulation of breast cancer progression and metastasis (1). The glycosaminoglycan chondroitin sulfate (CS) is present on the cell surface and in the extracellular matrix (ECM), including the glycocalyx. There is ample evidence for a pro-tumorigenic role for CS in the enhancement of cell proliferation, motility, and metastasis (2–5).

Chondroitin sulfate proteoglycans (CS-PGs) consist of a core protein and covalently attached CS chains. CS is a linear sulfated polymer of repeating disaccharide units of glucuronic acid (GlcA) and *N*-acetylgalactosamine (GalNAc) [-GlcA-GalNAc-]*_n_*. During the synthesis of the chondroitin backbone, multiple sulfotransferases catalyze the transfer of a sulfate group from 3’-phosphoadenosine 5’-phosphate, the universal donor in sulfation reactions, to their respective sulfation sites on GalNAc or GlcA residues in the CS chain. Based on the substrate preferences of chondroitin sulfotransferases identified to date, the biosynthetic scheme for CS-type sulfation can be separated into initial 4-*O*-sulfation and 6-*O*-sulfation pathways. In the initial step, the non-sulfated O unit [GlcA-GalNAc] serves as a common acceptor substrate for two types of sulfotransferases, chondroitin 4-*O*-sulfotransferases (CHST11 and CHST12) (6–8) and chondroitin 6-*O*-sulfotransferse-1 (CHST3), forming monosulfated A [GlcA-GalNAc(4-*O*-sulfate)] and C [GlcA-GalNAc(6-*O*-sulfate)] units, respectively. Subsequent sulfation of the A and C units also occurs *via* GalNAc 4-sulfate 6-*O*-sulfotransferase (CHST15) or CS-specific uronyl 2-*O*-sulfotransferase (UST), producing disulfated disaccharide E [GlcA-GalNAc(4,6-*O*-disulfate)] and D [GlcA(2-*O*-sulfate)-GalNAc(6-*O*-sulfate)] units, respectively (9). Of these sulfotransferases, it has been reported that the expression of CHST11 and CHST15 is upregulated in breast cancer cells (10). Moreover, the expression of *CHST11* has been correlated with breast-cancer progression (11). Specific sulfation patterns are hypothesized to underlie the distinct functional roles of CS not only under physiological conditions but also in tumor development and progression (12, 13).

Interestingly, it has been reported that the invasive activity of basal-like subtypes (MDA-MB-231 and BT-549 cells) is elevated by treatment with chondroitin sulfate E (CS-E), the major component of which is the E-unit (14). This result prompted us to hypothesize that there is a CS-E receptor in some cancer cells. We previously showed that N-cadherin functions as a receptor for CS-E in BT-549 cells, enhancing invasion activity by upregulating matrix metallopeptidase 9 *via* N-cadherin–catenin signaling. In another basal-like subtype of MDA-MB-231 cells, invasive activity was enhanced by treatment with CS-E; however, MDA-MB-231 cells do not express N-cadherin. In addition, CS-E had no effect on β-catenin-dependent transcription in MDA-MB-231 cells (14), suggesting that CS-E increases invasive activity mediated by non-canonical Wnt signaling. MDA-MB-231 cells express high levels of receptor tyrosine kinase-like orphan receptor 1 (ROR1), a receptor for non-canonical Wnt ligands such as WNT5A (15). ROR1 is associated with CS-dependent invasiveness of MDA-MB-231 cells. In addition, MDA-MB-231 cells express high levels of the Wnt signal modulator, Dickkopf-1 (DKK1), which binds directly to CS-E (14). Although DKK1 is a secreted inhibitor of β-catenin-dependent Wnt signaling, recent studies have shown that its elevated expression correlates with poor prognosis in a range of cancers (16). Here, we show that ROR1-dependent invasion activity is controlled by CSs in MDA-MB-231 cells. In addition, this study suggests that DKK1 exhibits tumor suppressor activity by blocking the tumor invasion activity of CS-E and by inhibiting β-catenin-dependent Wnt signaling.

## Materials and Methods

### Cell Culture and Stable Transfection

The human breast cancer cell line MDA-MB-231 (#92020424) was purchased from the European Collection of Cell Culture (ECACC) (Salisbury, UK). MCF7 cells (ATCC^®^ HTB-22™) were obtained from the American Type Culture Collection (ATCC). Both lines were cultured in Roswell Park Memorial Institute (RPMI) 1640 supplemented with 10% heat-inactivated fetal bovine serum (FBS), 100 units/ml penicillin, 100 μg/ml streptomycin, and 1% L-glutamine.

The expression plasmids [pcDNA3.1(+)-ROR1] were transfected into MCF7 cells using Lipofectamine 3000 (Thermo Fisher Scientific, Waltham, MA), according to the manufacturer’s instructions. Transfectants were cultured in the presence of 300 μg/ml G418. Colonies surviving in the presence of 300 µg/ml G418 were collected, and ROR1-expressing cell populations were enriched using 2 μg of anti-ROR1 antibody (Clone 4A5, Cat. No. 564464, BD Biosciences, Franklin Lakes, NJ, USA) and 1.5 mg of Dynabeads™ Protein G (Cat. No. 10003D, Invitrogen, Waltham, MA). The pooled ROR1-expressing clones were propagated for experiments.

### Plasmid Construction

A human ROR1 Flexi clone (FXC20341) was obtained from Kazusa Genome Technologies, Inc. (Chiba, Japan). For the expression of ROR1, polymerase chain reaction (PCR) was performed using the following primers and pF1KE3329-human ROR1 as a template: forward, 5’-GCTGGCTAGCGTTTA**ATG**CACCGGCCGCGCCGCCG-3’ [underline, homologous to the pcDNA3.1(+) vector sequence; bold, start codon]; reverse, 5’-GGTTTAAACGGGCCCTTACAGTTCTGCAGAAATCATAGATTCG-3’ [underline, homologous to the pcDNA3.1(+) vector sequence]. pCDNA3.1(+)-ROR1 was constructed using an In-Fusion^®^ HD cloning kit (TaKaRa Bio Inc., Shiga, Japan). Linearized pcDNA3.1(+)was generated by inverse PCR using the following primers and KOD One PCR master mix (TOYOBO, Osaka, Japan): forward, 5’-TAAACGCTAGCCAGC-3’, reverse, 5’-GGGCCCGTTTAAACC-3’.

### Real-Time PCR

Total RNA was isolated from cells using Sepasol^®^-RNA I Super G (Nacalai Tesque, Inc., Kyoto, Japan). For reverse transcription, 1 μg of total RNA was treated with Moloney murine leukemia virus reverse transcriptase (Promega, Madison, WI, USA) using random primers [nondeoxyribonucleotide mixture; pd(N)_9_] (Takara Bio Inc., Shiga, Japan). Quantitative real-time PCR was conducted using FastStart DNA Master Plus SYBR Green I in a LightCycler^®^ 96 (Roche Applied Science, Penzberg, Germany), according to the manufacturer’s protocol. The data processing was based on standard curves, and target-to-reference ratios were calculated using the relative quantification analysis module of the LigtCycler^®^ 96 software. The amplification efficiency was calculated based on the slope of the standard curve (target DNA was amplified with 90%–110% efficiency). Amplified DNA product was checked by post-PCR melting curve analysis. The housekeeping gene *GAPDH* was used as an internal control for quantification. Primers are listed in **Table 1**.

**Table 1:** Primers used for real-time PCR.

Gene Name	5’-primer		3’-primer	References	
hWNT1	5’-ACCGAGGCTGTCGAGAAACG-3’		5’-GCCGGTAGTCACACGTGCAG-3’	(14)	
hWNT2	5’-GCTCACCCCCGAGGTCAACT-3’		5’-CCTGGCTAATGGCACGCATC-3’	(14)	
hWNT3A	5’-ACAACAATGAGGCTGGG-3’		5’-ATCTCCGAGGCACTGTCATA-3’	(14)	
hWNT4	5’-CTCCACACTCGACTCCTTGC-3’		5’-CCGAAGAGATGGCGTACACG-3’	PrimerBank ID156630997c2	
hWNT5A	5’-TCGACTATGGCTACCGCTTTG-3’		5’-CACTCTCGTAGGAGCCCTTG-3’	PrimerBank ID3371506361c3	
hWNT5B	5’-CATGGCCTACATAGGGGAGG -3’		5’-CTGTGCTGCAATTCCACCG-3’	PrimerBank ID17402920c2	
hWNT6	5’-GGCAGCCCCTTGGTTATGG-3’		5’-CTCAGCCTGGCACAACTCG-3’	PrimerBank ID53729353c1	
hWNT7A	5’-CTGTGGCTGCGACAAAGAGAA-3’		5’-GCCGTGGCACTTACATTCC-3’	PrimerBank ID34328912c1	
hWNT11	5’-GGAGTCGGCCTTCGTGTATG-3’		5’-GCCCGTAGCTGAGGTTGTC-3’	PrimerBank ID17017973c1	
hDKK-1	5’-GTGCGCAGAGGACGAGGAGT-3’		5’-GTGACGCATGCAGCGTTTTC-3’	(14)	
hROR1	5’-CAGTCAGTGCTGAATTAGTGCC-3’		5’-TCATCGAGGGTCAGGTAAGAAT-3’	PrimerBank ID 134152685c1	
hROR2	5’-GTGCGGTGGCTAAAGAATGAT-3’		5’-ATTCGCAGTCGTGAACCATATT-3’	PrimerBank ID 317008621c2	
hCHST11	5’-AAACGCCAGCGGAAGAA-3’		5’-GGGATGGCAGAGTGAGTAGA-3’	(7)	
hCHST15	5’-TCGTGTGGACAGTAAGCAGAT-3’	5’-TGTAAGAAGCCATTACCAAGGTC-3’		PrimerBank ID 311893349c1

### Knockdowns

Cells were transfected with Silencer^®^ Select siRNAs targeting *ROR1* (assay ID s9755), *WNT5A* (assay ID: s14873), *CHST11* (assay ID: s27032 and s27033), *CHST15* (assay IDs: s28015 and s28017), and *DKK1* (assay ID: s22721) purchased from Thermo Fisher Scientific. Forty-two hours after transfection, cells were subjected to invasion and migration assays.

### Invasion and Migration Assays

Invasion was assessed using Corning^®^ BioCoat™ Matrigel^®^ invasion chambers, according to the manufacturer’s instructions. Cells were collected by treatment with trypsin/EDTA, washed twice with phosphate-buffered saline (PBS), pelleted, and resuspended in medium containing 0.2% fetal bovine serum or 0.1% bovine serum albumin. Cells (5 × 10^4^/ml) were incubated in the presence or absence of 50 μg/ml CS-E (Seikagaku Corporation, Tokyo, Japan) or 10 μM JNK inhibitor SP600125 (Cat. No. 129-56-6, Cayman Chemical Company, Ann Arbor, MI) for 20 min at 25°C, added to the upper chamber, and allowed to invade for 22 h at 37°C in a CO_2_ incubator. Complete medium containing 10% fetal bovine serum or 200 ng/ml recombinant human/mouse Wnt-5A (Cat. No. 645-WN/CF) was placed in the bottom well as chemoattractant. Migration was assessed using Corning^®^ BioCoat™ control inserts according to the manufacturer’s instructions. Cells (5 × 10^4^/ml) were prepared as described above.

### Immunoblotting

Cells were treated with 50 μg/ml CS-E or CS-A (Seikagaku Corporation, Tokyo, Japan), 50 μg/ml synthesized hexasaccharides GalNAc(4-*O*-sulfate,6-*O*-sulfate)-GlcA-GalNAc(4-*O*-sulfate,6-*O*-sulfate)-GlcA-GalNAc(4-*O*-sulfate,6-*O*-sulfate)-GlcA-*O-p*-methoxyphenyl (E-E-E 6-mer), or GalNAc-GlcA-GalNAc-GlcA-GalNAc-GlcA-O-p-methoxyphenyl (O-O-O 6-mer) for the indicated times. Cells were solubilized in M-PER (Thermo Fisher Scientific) containing a protease inhibitor cocktail (Nacalai Tesque, Inc., Kyoto, Japan) and 10 μM proteasome inhibitor (MG132; PEPTIDE Institute. Inc., Osaka, Japan) for 30 min on ice. Lysates were centrifuged at 16,500×*g* for 15 min. Proteins were separated on Bullet PAGE precast gels, 5-15% (Nacalai Tesque, Inc.), transferred to polyvinylidene fluoride (PVDF) membranes, and incubated overnight with primary antibodies against phosphor-c-Jun N-terminal kinase (JNK) (T183/Y185) (clone 81E11, Cat. No. 4668S, Cell Signaling Technology, Danvers, MA, USA), actin (clone AC-40, Cat. No. A3853, Sigma-Aldrich, St. Louis, MO), Ror1 (clone D6T8C, Cat. No. 16540S, Cell Signaling Technology), phospho-cortactin (Y421) (Cat. No. 4569S, Cell Signaling Technology), and cortactin (clone H222, Cat. No. 3503S; Cell Signaling Technology).

### Flow Cytometry

Cells (1×10^6^) were fixed with PBS containing 4% paraformaldehyde on ice for 30 min. After washing with PBS, cells were incubated with PBS containing 2% BSA on ice for 30 min and then stained with anti-ROR1 antibody (dilution ratio, 1:100) (Clone 4A5, Cat. No. 564464, BD Biosciences, Franklin Lakes, NJ, USA) on ice. After 1 h, cells were washed and incubated with mouse IgG2b antibody conjugated with Alexa™488 (dilution ratio, 1:400) (Thermo Fisher Scientific) on ice for 1 h. Cells were analyzed using a BD Accuri™ C6 flow cytometer (BD Biosciences).

### Disaccharide Analysis of CSs From Human Breast Cancer Cell Lines

CSs isolated and purified from human breast cancer MDA-MB-231 and MCF7 cells were analyzed as previously described (6, 7, 17–19).

### Biacore Analysis

Real-time binding was assessed as described previously (19), using Biacore X100 (Cytiva, Tokyo, Japan). Recombinant human ROR1-Fc chimera (Cat. No. 9490-RO, R&D Systems) was immobilized on a CM5 series chip (Cytiva) using the amine-coupling method. ROR1 injection was stopped when the surface plasmon resonance reached ~ 3,200 RU. For ROR1 binding assays, WNT5A (0, 0.038, 0.075, 0.15, 0.30, and 0.60 μM), WNT5A/CS-E (0, 0.036/0.25, 0.071/0.50, 0.15/1.0, 0.29/2.0, and 0.57/4.0 μM), recombinant human DKK1 (Cat. No. 5439-DK/CF, R&D Systems) (0, 0.038, 0.075, 0.15, and 0.3 μM), or DKK1/CS-E (0, 0.038/0.28, 0.075/0.55, 0.15/1.1, and 0.3/2.1 μM) were sequentially injected at a flowrate of 30 μl/min for 120 s at 25°C; the dissociation time was set for 130 s. Binding reactions were performed in 50 mM Tris–HCl buffer (pH 7.5).

### Statistical Analysis

Data are expressed as means ± one standard deviation. Statistical significance was determined using Student’s *t-*test or one-way ANOVA, with Tukey’s honestly significant difference (HSD) test.

## Results

### Invasion-Promoting Activity of CS-E Is Mediated by WNT5A/ROR1/JNK Signaling

**Figure 1:**
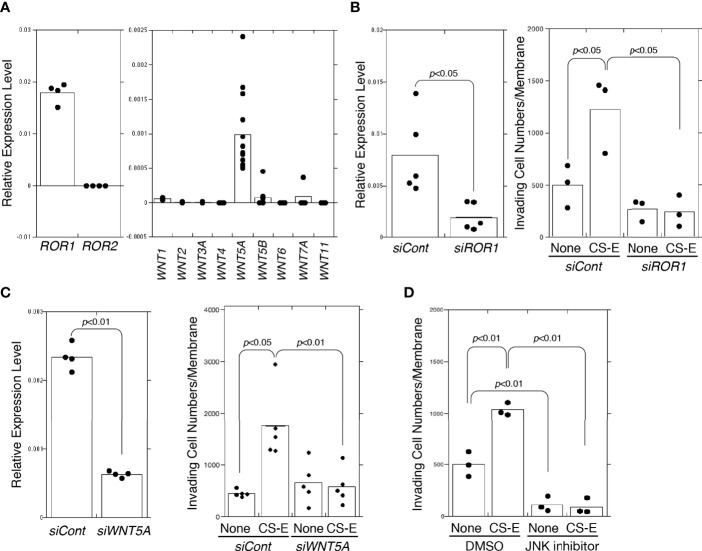
CS-E-elicited invasiveness *via* WNT5A-ROR1-JNK signaling. **(A)** Expression levels of ROR1 (n=4), ROR2 (n=4), WNT1 (n=3), WNT2 (n=3), WNT3A (n=3), WNT4 (n=4), WNT5A (n=13), WNT5B (n=8), WNT6 (n=4), WNT7A (n=4), and WNT11 (n=4) in MDA-MB-231 cells measured by qPCR. **(B)** *ROR1* expression after *ROR1* knockdown (*siROR1*) (n=5) or control cells (*siCont*) (n=5) measured by qPCR. Invasiveness of *siROR1*-treated cells (*siROR1*) (n=3) or control cells (*siCont*) (n=3) was measured in the absence or presence of 50 μg/ml CS-E. Data were analyzed using Student’s *t*-test. **(C)** Left graph: *WNT5A* mRNA expression decreased at the mRNA level following siRNA-induced knockdown in MDA-MB-231 by qPCR (n=4). Expression data were normalized to those of *GAPDH*. Right graph: invasiveness of *WNT5A*-knockdown (siWNT5A) and control cells (siCont) measured in the absence or presence of 50 μg/ml of CS-E (n=5). Data were analyzed using Student’s *t*-test. **(D)** MDA-MB-231 cells treated with DMSO or 10 μM JNK inhibitor SP600125, assessed for invasiveness in the absence or presence of 50 μg/ml CS-E.

We have shown that the invasive activity of basal-like subtypes (MDA-MB-231 and BT-549 cells) is elevated by treatment with chondroitin sulfate E (CS-E) (13). Because MDA-MB-231 cells express mRNAs for the receptors in the canonical Wnt signaling pathway, LRP5 and LRP6, at low levels (13), we hypothesized that the receptors for the non-canonical Wnt signaling pathway, ROR1 and ROR2, are targeted by CS-E in MDA-MB-231 cells. Quantitative real-time PCR (qRT-PCR) indicated that ROR1 was predominantly expressed in MDA-MB-231 cells (**Figure 1A**). Because it is known that Ror1 is a receptor for WNT5A and WNT5B (20), their gene expression levels were examined (**Figure 1A**). Among the Wnt ligands examined, *WNT5A* mRNA was expressed at the highest level in MDA-MB-231 cells. To determine whether ROR1 is involved in invasion activity enhanced by treatment with CS-E, *ROR1* was knocked down. The invasion potential of MDA-MB-231 cells was significantly upregulated in response to CS-E, whereas MDA-MB-231 cells became less responsive to CS-E after *ROR1* knockdown (**Figure 1B**). Next, we examined the effect of *WNT5A* knockdown on invasiveness elicited by CS-E (**Figure 1C**) but did not observe an increase. As WNT5A signals are transduced through RORs to activate the c-Jun N-terminal kinase (JNK) pathway (21–23), we determined whether an inhibitor of JNK, SP600125, altered invasiveness in MDA-MB-231 cells (**Figure 1D**). As expected, CS-E did not promote invasiveness in the presence of JNK inhibitor. These results suggest that CS-E enhances the invasive activity of MDA-MB-231 cells through the WNT5A−ROR1−JNK signaling axis.

### CSs activate JNK in a Sulfation Pattern-Dependent Manner

**Figure 2:**
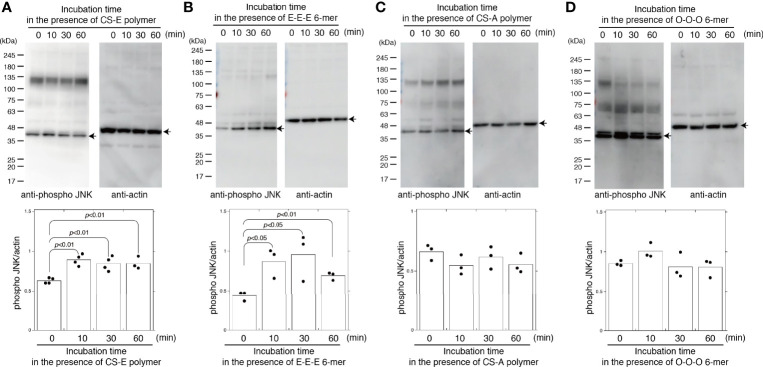
CS-E and chemically-synthesized E unit-containing hexasaccharides specifically activate JNK. MDA-MB-231 cells were treated with 50 μg/ml of CS-E polymers **(A)**, 50 μg/ml chemically synthesized hexasaccharides consisting of three E units (E-E-E 6 mer) **(B)**, 50 μg/ml of CS-A polymers **(C)**, or 50 μg/ml chemically synthesized hexasaccharides consisting of three O units (O-O-O 6 mer) **(D)** at the indicated times. Phosphorylated JNK and actin levels were assessed by immunoblotting (top of each panel) and quantified by densitometry (bottom of each panel) (n≧3). Representative protein bands are indicated by arrows. Statistical significance was assessed using Student’s *t*-test.

Treatment with CS-E polymers for 10 min significantly elevated JNK phosphorylation (**Figure 2A**). The CS-E polymers used in this study contained 72% E, 18.4% A, 8.7% C, and 6.6% O units. To determine whether the E-disaccharide unit is required to activate JNK, we used chemically synthesized hexasaccharide sequences, including the E-E-E-containing sequence GalNAc(4-*O*-sulfate,6-*O*-sulfate)-GlcA-GalNAc(4-*O*-sulfate,6-*O*-sulfate)-GlcA-GalNAc(4-*O*-sulfate,6-*O*-sulfate)-GlcA-*O-p*-methoxyphenyl (E-E-E 6-mer). The E-E-E 6-mer also activated JNK in MDA-MB-231 cells (**Figure 2B**). These results suggest that the E units in the CS chains play an important role in the activation of JNK.

As described in *Introduction*, CS exhibits highly diverse structural variations that contribute to its functional diversity. Therefore, we determined whether CS-E specifically elicits JNK phosphorylation. Chondroitin sulfate A (CS-A) polymers used in this study contained 74.5% A units, 23.7% C units, 1.9% O units, 1.8% D units, and 0% E units. CS-A polymers did not activate JNK (**Figure 2C**). In addition, chemically synthesized non-sulfated hexasaccharides GalNAc-GlcA-GalNAc-GlcA-GalNAc-GlcA-*O-p*-methoxyphenyl (O-O-O 6-mer) did not activate JNK (**Figure 2D**). These results suggest that CS controls JNK activation in a sulfation-pattern-dependent manner.

### E Unit-Containing CS Chains Enhance Invasiveness

**Figure 3:**
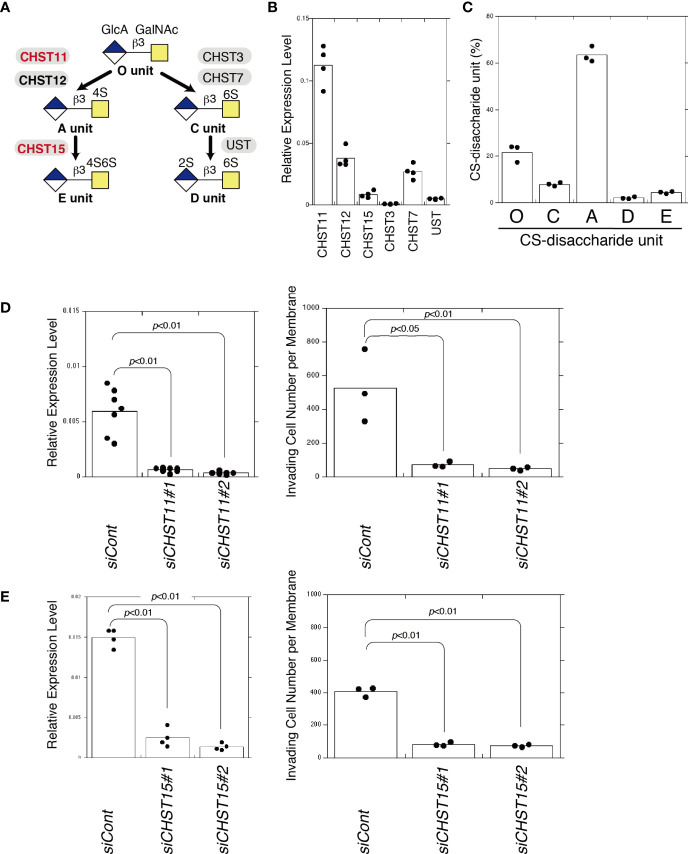
Decreased E unit biosynthesis decreases invasiveness. **(A)** Diagram of sulfation pathways. The C4-position of the GalNAc residue in the O unit is sulfated by CHST11 and CHST12 to form an A unit. Subsequently, the A unit is converted to an E unit by CHST15. **(B)** mRNA expression levels of chondroitin sulfotransferases, *CHST11*, *CHST12*, *CHST15*, *CHST3*, *CHST7*, and *UST* in MDA-MB-231 cells measured by qPCR (*n=4*). **(C)** Composition of CS-disaccharide units of sulfated CS chains isolated from MDA-MB-231 cells analyzed using HPLC (n=3). **(D)** *CHST11* mRNA expression decreased following two kinds of siRNA-induced knockdown (*siCHST11#1* and *siCHST11#2*) in MDA-MB-231 cells, measured using qPCR (n=7). Expression data were normalized to those of *GAPDH*. Right graph: Invasiveness of *CHST11* knocked down cells (*siCHST11#1* or *siCHST11#2*) (n=3) or control cells (*siCont*) (n=3). **(E)** *CHST15* mRNA expression decreased at the mRNA level following knockdowns by *siCHST15#5* and *siCHST15#7* in MDA-MB-231 cells measured using qPCR (n=4). Expression data were normalized to those of *GAPDH*. Right graph, invasiveness of *CHST15* knocked down cells (*siCHST15#1* or *siCHST15#2*) (n=3) or control cells (*siCont*) (n=3). Data were analyzed using the Tukey–Kramer multiple comparison.

As shown in **Figure 3A**, the disaccharide unit of CS, GlcA-GalNAc, is sulfated by the indicated sulfotransferases. The E unit is synthesized by CHST11 (carbohydrate sulfotransferase 11, C4ST-1) and CHST15 (carbohydrate sulfotransferase 15, GALNAC4S-6ST). The expression levels of these sulfotransferases in MDA-MB-231 cells were examined (**Figure 3B**). The CS chains produced in MDA-MB-231 cells contained 63.5% A, 21.8% O, 7.9% C, 4.5% E, and 2.2% D units (**Figure 3C**). To downregulate the expression of the A and E units, *CHST11* was knocked down; this strongly decreased the invasiveness of MDA-MB-231 cells (**Figure 3D**). When only E units were decreased by CHST15 knockdown, the invasiveness was suppressed (**Figure 3E**). These results suggest that a specific sulfation pattern, the E unit, is associated with the high invasive potential of MDA-MB-231 cells.

### Increased Invasiveness Elicited by Expression of ROR1 Is Diminished by *CHST15* Knockdown in MCF7 Cells

**Figure 4:**
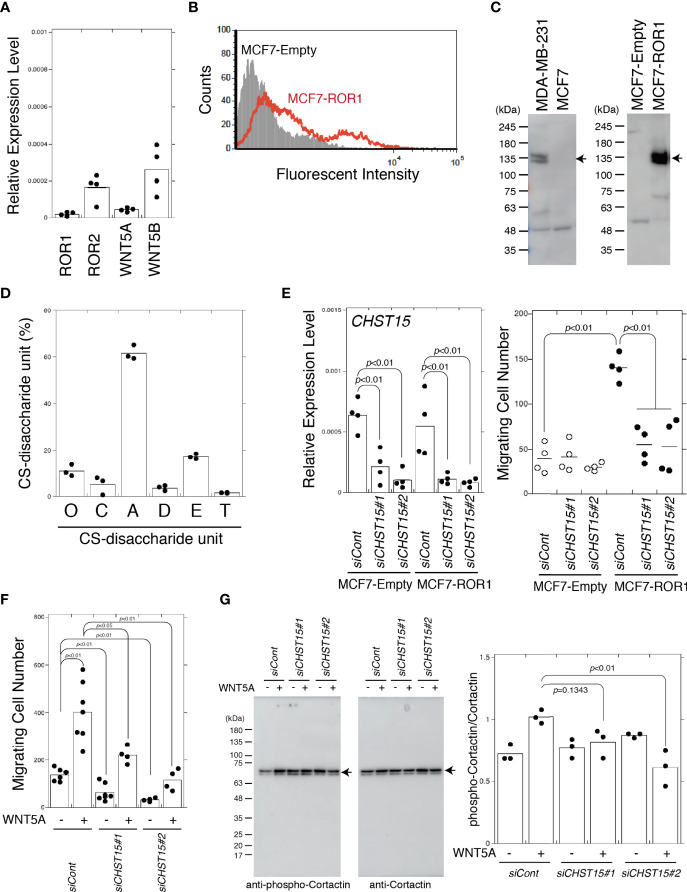
MCF7 cells acquire invasiveness by expression of ROR1 and lose it by knockdown of *CHST15*. **(A)** mRNA expression levels of chondroitin sulfotransferase genes, *ROR1*, *ROR2*, *WNT5A*, and *WNT5B* in MDA-MB-231 cells measured by qPCR (*n=4*). **(B)** ROR1 surface expression by MCF7-empty cells (shaded area) and MCF7-Ror1 cells (red line) measured by flow cytometry. **(C)** Left, ROR1 expression in MDA-MB-231 and MCF7 cells measured by immunoblotting. Right, ROR1 expression MCF7 cells stably transfected with an empty vector (MCF7-Empty) or an ROR1 expression plasmid (MCF7-ROR1) analyzed by immunoblotting. **(D)** CS-disaccharide composition of sulfated CS chains from MCF7 cells analyzed using HPLC (n=3). **(E)** Left, *CHST15* mRNA expression following siRNA-induced knockdown (*siCHST15#1* or *siCHST15#2*) in MCF7-empty cells and MCF7-ROR1 cells measured using qPCR (n=4). Expression data were normalized to those of *GAPDH*. Right, migration of *CHST15* knocked down cells (*siCHST15#1* or *siCHST15#2*) (n=4) and control cells (*siCont*) (n=4) measured using a Transwell assay. **(F)** *CHST15* knocked down MCF7-ROR1 cells (*siCHST15#1* or *siCHST15#2*) or control cells (*siCont*) serum-starved and treated with (+) or without (−) 200 ng/ml of WNT5A. **(G)** *CHST15* knocked down MCF7-ROR1 cells (*siCHST15#1* or *siCHST15#2*) or control cells (*siCont*) treated with or without 100 ng/ml of WNT5a for 5 min, lysed, and subjected to immunoblotting using anti-phospho-cortactin and anti-cortactin antibodies. Right graph, relative levels of phosphorylated cortactin, standardized against total cortactin (n=4). Statistical significance was assessed using Student’s *t*-test.

MCF7 is a less-aggressive, non-invasive cell line that is normally considered to have low metastatic potential (24). MCF7 cells expressed low levels of ROR2, WNT5A, and WNT5B (**Figure 4A**). Endogenous ROR1 (**Figures 4A, C**) and DKK1 proteins (13) were not detected in MCF7 cells, so ROR1-expressing cells were generated. ROR1 surface expression in MCF7 cells stably transfected with an empty vector (MCF7-empty cells) or stably expressing ROR1 (MCF7-ROR1 cells) was confirmed by flow cytometry (**Figure 4B**). Immunoblotting showed that MCF7-ROR1 cells expressed ROR1 at high levels (**Figure 4C**). As shown in **Figure 4D**, MCF7 cells produced CSs containing 20% E. Next, we examined whether the E unit was required for ROR1-mediated migration activity using MCF7-ROR1 cells. The migration potential of MCF7 cells was increased by ROR1 expression. This increase was attenuated by *CHST15* knockdown (**Figure 4E**). In addition, enhanced migration stimulated by treatment with WNT5A was suppressed by knockdown of *CHST15* (**Figure 4F**). Hasan et al. reported that WNT5A stimulates ROR1-dependent cortactin phosphorylation and enhances cell migration (25). We found that CS-E increased the phosphorylation of cortactin by WNT5A and that *CHST15* knockdown inhibited cortactin phosphorylation (**Figure 4G**). These results suggest that CSs regulate ROR1-mediated migration of breast cancer cells.

### CS-E Directly Interacts With ROR1 in the Presence of Either WNT5A or DKK1

**Figure 5:**
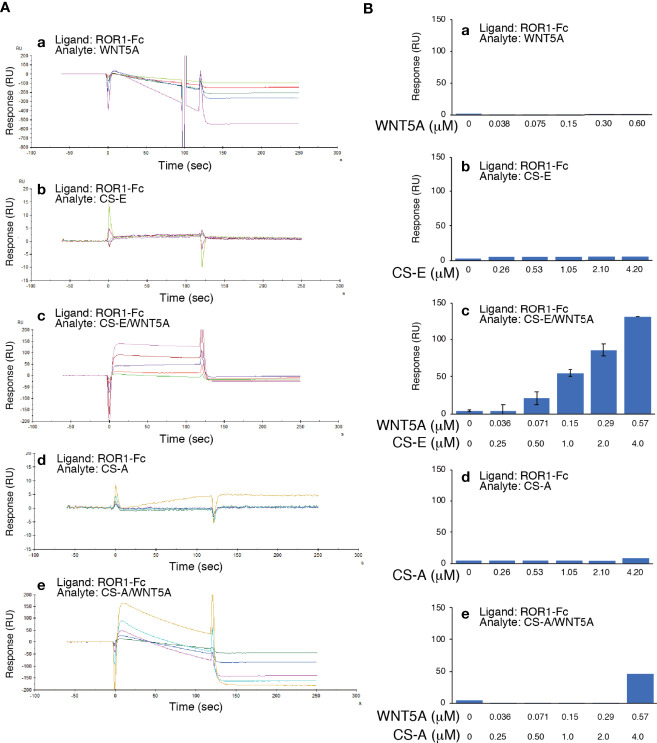
WNT5A binds ROR1 in the presence of CS-E. **(A)** Raw sensor grams. ROR1 was immobilized in a flow cell of a CM5 sensor chip. WNT5A (a), CS-E (b), WNT5A pre-mixed with CS-E (c), CS-A (d), and WNT5A pre-treated with CS-A (e) were used as analytes. **(B)** Response-unit quantification of binding.

By surface plasmon resonance (SPR) analysis, neither WNT5A nor CS-E bound to ROR1 (**Figures 5A, B**). In contrast, CS-E pre-mixed with WNT5A at a molar ratio of 7:1 did bind ROR1 (**Figures 5A, B**).

**Figure 6:**
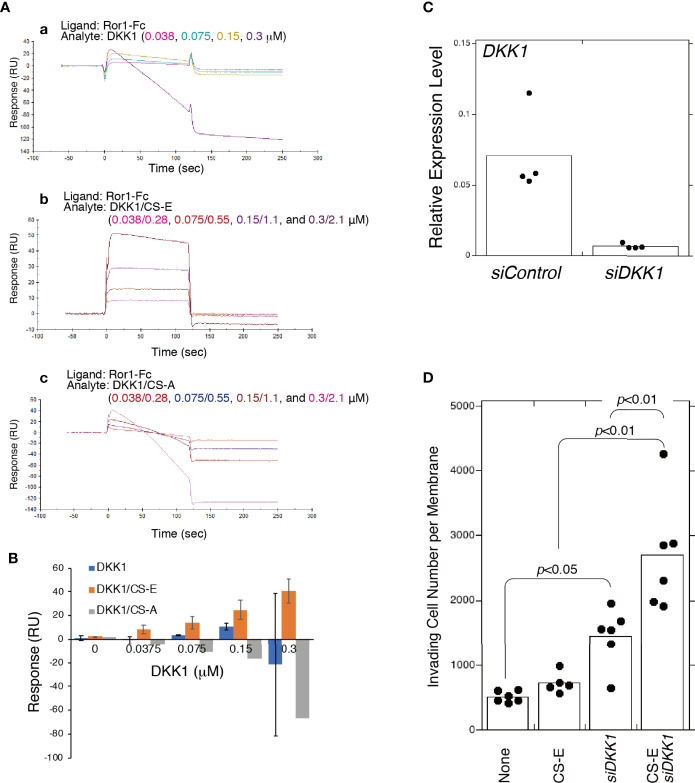
CS-E-elicited invasiveness is enhanced by the absence of DKK1. **(A)** Raw sensor grams. ROR1 was immobilized in a flow cell of a CM5 sensor chip. DKK1 alone **(a)**, DKK1 premixed with CS-E at a 1:7 molar ratio **(b)**, and DKK1 premixed with CS-A at a 1:7 molar ration **(c)** were used as analytes. **(B)** Response-unit quantification of binding. **(C)** *DKK1* mRNA expression in MDA-MB-231 cells transfected with *siDKK1* or control siRNA (*siCont*) measured using qPCR (n=4). Expression data were normalized to those of *GAPDH*. **(D)** Invasiveness of *DKK1* knocked down MDA-MB-231 cells (*siDKK1*) or control cells (*siCont*) treated with or without CS-E (n>5). Data were analyzed using a Tukey–Kramer multiple comparison.

As it has been previously shown that DKK1 binds to CS-E (13), we hypothesized that DKK1 was able to bind to ROR1 by forming a complex with CS-E. DKK1 is known to bind to LDL receptor-related proteins 5 and 6 (LRP5/6), which function as coreceptors in the canonical WNT signaling pathway, but no direct interaction between DKK1 and ROR1 has been shown. SPR indicated that DKK1 weakly bound to ROR1 in the concentration range of 0.0375 to 0.15 μM (**Figures 6A, B**). Binding of DKK1 to ROR1 was not reproducibly observed at 0.3 μM DKK1. CS-E was premixed with DKK1 at a molar ratio of 7:1, after which it was bound to ROR1 in a concentration-dependent manner, while DKK1 did not bind to ROR1 in the presence of CS-A (**Figures 6A, B**). These results suggested that DKK1 binds to ROR1 in the presence of CS-E.

We next examined whether CS-E increased the invasiveness of MDA-MB-231 cells by forming a complex with DKK1. Unexpectedly, *DKK1* knockdown enhanced CS-E-elicited invasiveness (**Figure 6D**). These results suggest that ROR1-dependent invasion signaling is blocked by binding of the DKK1/CS-E complex to ROR1 and that DKK1 exerts anti-tumorigenic effects by blocking the tumor invasion activity of CS-E as well as by inhibiting β-catenin-dependent Wnt signaling.

## Discussion

### ROR1 as a Potential CS Target

Focusing on the roles of CS in regulating cancer hallmark capabilities, we show here the involvement of ROR1, known as a receptor for WNT5A, in CS-E-mediated upregulation of invasion activity in MDA-MB-231 cells (14). Particular attention has been paid to the functional and clinical significance of ROR1 in many malignancies, including breast cancer. ROR1 is present in breast cancer specimens, but not in normal breast tissues (26), and high expression of ROR1 in breast cancer is associated with aggressive phenotype (27, 28). Several therapeutic strategies targeting ROR1 have been developed and evaluated in clinical trials (15).

**Figure 7:**
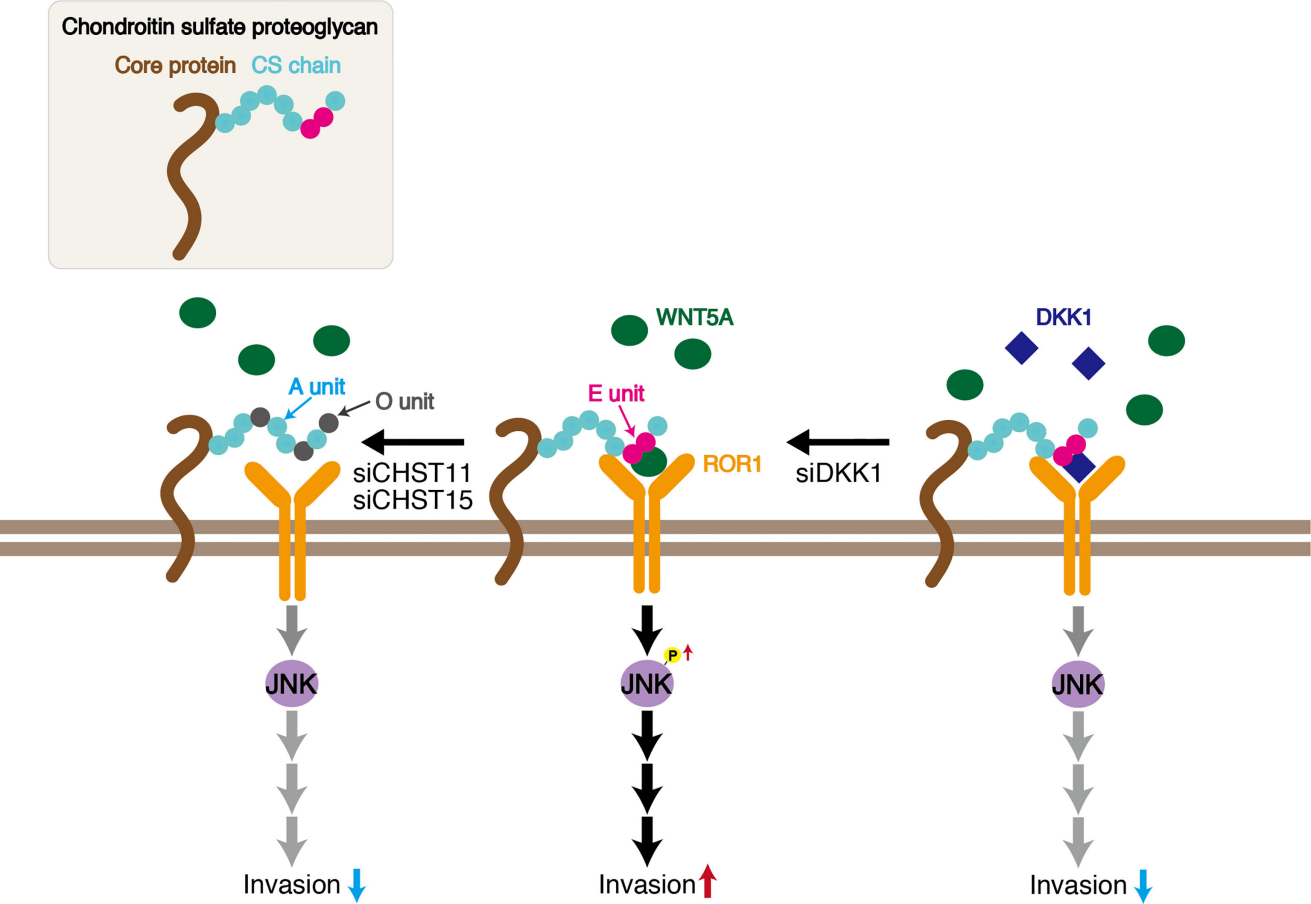
Schematic of CS-E enhancement of invasive activity of the triple-negative breast cancer MDA-MB-231 cell line. CS chains bind WNT5A and ROR1 through E units, signaling cancer cells to activate JNK1. Decreasing E units by knockdown of *CHST11* and *CHST15* inhibits WNT5A−ROR1−JNK signaling. DKK1 suppresses CS tumor promoting activity by binding to E units.

ROR1 is a transmembrane receptor that contains extracellular, transmembrane, and cytoplasmic domains. The extracellular region includes immunoglobulin-like, cysteine-rich (CRD), and Kringle domains. Although ligands for ROR1 have not been identified for many years, it is now known that ROR1 is a receptor for WNT5A/B and WNT16, with WNT5A as the primary ligand (15, 29, 30). The CRD of ROR1 is thought to interact with WNT5A (31, 32). Although Fukuda et al. showed *in vitro* binding of ROR1 to WNT5A using immunoprecipitated WNT5A expressed in CHO cells (27), the possibility that CSs mediate binding between ROR1 and WNT5A cannot be excluded. We analyzed the direct interaction between recombinant ROR1 and WNT5A using Biacore, but unexpectedly, we observed no binding in the absence of CS (**Figure 5**). The cell-surface chondroitin sulfate proteoglycan functions as a coreceptor in WNT5A−ROR1 signaling (**Figure 7**). Further studies are needed to clarify whether CS-E universally act on WNT5A-ROR1 signaling using other ROR1-positive and estrogen-negative cell lines.

### CS Sulfation and Cell Signaling in Cancer

Extensive evidence supports the importance of tumor-associated CSs in promoting aggressive and metastatic behavior of malignant cells by engaging transmembrane receptors (4, 33). In addition, it has been reported that cancer-cell surface CS chains facilitate downstream signal transduction through specific binding to adhesion molecules, including selectins and N-cadherin (14, 34). CS activity is governed by specific sulfation patterns. As mentioned in *Introduction*, CS is modified by sulfation by multiple sulfotransferases, including CHST11 and CHST15, which are involved in the synthesis of E units and are upregulated in breast cancer cells (4, 10). Although E units have low abundance in mammals, they play a critical role in regulating tumor progression and metastasis (35, 36). We have previously reported that CSs rich in E units (CS-E) utilize N-cadherin as a receptor and control β-catenin signaling *via* N-cadherin in the human basal-like breast cancer cell line BT-549. In this study, we showed that the E unit is required for the binding of WNT5A to ROR1 (**Figure 7**). CSs rich in A-units (CS-A) did not mediate the interaction between WNT5A and ROR1. A decline in the expression levels of CHST11 and CHST15, which are involved in the synthesis of E units, suppresses the invasive activity of MDA-MB-231 cells (**Figure 7**). Thus, CS-E likely contributes to breast cancer malignancy. Further studies are needed to elucidate whether cancer-cell-derived CS-E promotes tumor growth and metastasis *in vivo*.

Changes in the degree of sulfation and/or the pattern of CSs and the expression level of Chn sulfotransferases are associated with cancer; these changes have been proposed as cancer biomarkers (37). Furthermore, the degree of sulfation and/or the pattern of CSs exhibit person-to-person variations. In addition, the same cancers at different stages are associated with different sulfation changes (38). Thus, identifying tumor subtypes using CS sulfation patterns may have potential applications in patient stratification for therapy. CS sulfation patterns are thus key components in future diagnostic and therapeutic strategies and provide novel targets for improved and personalized cancer therapy (39).

### Another CS-E Partner Protein, DKK1

DKK1 was originally characterized as a secreted inhibitor of canonical WNT signaling. It binds to LRP5/6 coreceptors with high affinity and blocks β-catenin-dependent WNT signaling (40). MDA-MB-231 cells express high levels of DKK1 but LRP5/6 at low levels (14). We have previously shown that DKK1 binds to CS-E in a sulfation-dependent manner (14). Furthermore, recent studies have shown that elevated DKK1 expression contributes to tumor growth and poor prognosis in a range of cancers, suggesting a role in tumor aggressiveness independent of WNT signaling (16). Cytoskeleton-associated protein 4 (CKAP4) is a novel DKK1 receptor that binds to DKK1 to activate AKT signaling and enhance cancer cell proliferation (41). These findings prompted us to hypothesize that DKK1 binds to an unidentified receptor in the presence of CS-E to enhance the invasive potential of MDA-MB-231 cells. However, knockdown of *DKK1* increased invasive activity (**Figures 6**, **7**). Interestingly, invasiveness enhanced by exogenously added CS-E was further increased by knockdown of *DKK1* (**Figure 6**). These results suggested that DKK1 suppresses the tumor-promoting activity of CS-E by binding to CS-E. The ability of CS-E to function as a tumor promoter likely depends on the expression level of CS-E partner proteins such as N-cadherin, ROR1, and DKK1.

## Data Availability Statement

The raw data supporting the conclusions of this article will be made available by the authors, without undue reservation.

## Author Contributions

SN performed research and analyzed data. JT synthesized hexasaccharides. SN and HK wrote the manuscript. SN and HK conceived and designed the study. HK coordinated the study. All authors contributed to the article and approved the submitted version.

## Funding

This work was supported in part by Grants-in-Aid for Scientific Research on Innovative Areas 23110003 (to HK) and Scientific Research (B) 25293014 and 20H03386 (to HK) and (C) 17K07353 and 21K06089 (to SN), and by the Support Program for Strategic Research Foundation at Private Universities 2012–2016 (to HK) from the Ministry of Education, Culture, Sports, Science, and Technology, Japan.

## Conflict of Interest

The authors declare that the research was conducted in the absence of any commercial or financial relationships that could be construed as potential conflicts of interest.

## Publisher’s Note

All claims expressed in this article are solely those of the authors and do not necessarily represent those of their affiliated organizations, or those of the publisher, the editors and the reviewers. Any product that may be evaluated in this article, or claim that may be made by its manufacturer, is not guaranteed or endorsed by the publisher.

## References

[1] Kang H Wu Q Sun A Liu X Fan Y Deng X. Cancer Cell Glycocalyx and Its Significance in Cancer Progression. Int J Mol Sci (2018) 19:2484. doi: 10.3390/ijms19092484 PMC6163906 30135409

[2] Yang J Price MA Neudauer CL Wilson C Ferrone S Xia H. Melanoma Chondroitin Sulfate Proteoglycan Enhances FAK and ERK Activation by Distinct Mechanisms. J Cell Biol (2004) 165:881–91. doi: 10.1083/jcb.200403174 PMC2172406 15210734

[3] Yang J Price MA Li GY Bar-Eli M Salgia R Jagedeeswaran R. Melanoma Proteoglycan Modifies Gene Expression to Stimulate Tumor Cell Motility, Growth, and Epithelial-to-Mesenchymal Transition. Cancer Res (2009) 69:7538–47. doi: 10.1158/0008-5472.CAN-08-4626 PMC2762355 19738072

[4] Cooney CA Jousheghany F Yao-Borengasser A Phanavanh B Gomes T Kieber-Emmons AM. Chondroitin Sulfates Play a Major Role in Breast Cancer Metastasis: A Role for CSPG4 and CHST11 Gene Expression in Forming Surface P-Selectin Ligands in Aggressive Breast Cancer Cells. Breast Cancer Res (2011) 13:R58. doi: 10.1186/bcr2895 21658254 PMC3218947

[5] Willis CM Kluppel M. Chondroitin Sulfate-E is a Negative Regulator of a Pro-Tumorigenic Wnt/beta-Catenin-Collagen 1 Axis in Breast Cancer Cells. PloS One (2014) 9:e103966. doi: 10.1371/journal.pone.0103966 25090092 PMC4121171

[6] Nadanaka S Ishida M Ikegami M Kitagawa H. Chondroitin 4-O-Sulfotransferase-1 Modulates Wnt-3a Signaling Through Control of E Disaccharide Expression of Chondroitin Sulfate. J Biol Chem (2008) 283:27333–43. doi: 10.1074/jbc.M802997200 18667431

[7] Nadanaka S Kinouchi H Taniguchi-Morita K Tamura J Kitagawa H. Down-Regulation of Chondroitin 4-O-Sulfotransferase-1 by Wnt Signaling Triggers Diffusion of Wnt-3a. J Biol Chem (2011) 286:4199–208. doi: 10.1074/jbc.M110.155093 PMC3039320 21123170

[8] Nadanaka S Kinouchi H Kitagawa H. Histone Deacetylase-Mediated Regulation of Chondroitin 4-O-Sulfotransferase-1 (Chst11) Gene Expression by Wnt/beta-Catenin Signaling. Biochem Biophys Res Commun (2016) 480:234–40. doi: 10.1016/j.bbrc.2016.10.035 27751852

[9] Mikami T Kitagawa H. Biosynthesis and Function of Chondroitin Sulfate. Biochim Biophys Acta (2013) 1830:4719–33. doi: 10.1016/j.bbagen.2013.06.006 23774590

[10] Iida J Dorchak J Clancy R Slavik J Ellsworth R Katagiri Y. Role for Chondroitin Sulfate Glycosaminoglycan in NEDD9-Mediated Breast Cancer Cell Growth. Exp Cell Res (2015) 330:358–70. doi: 10.1016/j.yexcr.2014.11.002 25445787

[11] Hazan RB Phillips GR Qiao RF Norton L Aaronson SA. Exogenous Expression of N-Cadherin in Breast Cancer Cells Induces Cell Migration, Invasion, and Metastasis. J Cell Biol (2000) 148:779–90. doi: 10.1083/jcb.148.4.779 PMC2169367 10684258

[12] Kitagawa H. Using Sugar Remodeling to Study Chondroitin Sulfate Function. Biol Pharm Bull (2014) 37:1705–12. doi: 10.1248/bpb.b14-00613 25366475

[13] Nadanaka S Kitagawa H. Exostosin-Like 2 Regulates FGF2 Signaling by Controlling the Endocytosis of FGF2. Biochim Biophys Acta Gen Subj (2018) 1862:791–9. doi: 10.1016/j.bbagen.2018.01.002 29305908

[14] Nadanaka S Kinouchi H Kitagawa H. Chondroitin Sulfate-Mediated N-Cadherin/Beta-Catenin Signaling is Associated With Basal-Like Breast Cancer Cell Invasion. J Biol Chem (2018) 293:444–65. doi: 10.1074/jbc.M117.814509 PMC5767853 29183998

[15] Zhao Y Zhang D Guo Y Lu B Zhao ZJ Xu X. Tyrosine Kinase ROR1 as a Target for Anti-Cancer Therapies. Front Oncol (2021) 11:680834. doi: 10.3389/fonc.2021.680834 34123850 PMC8193947

[16] Kagey MH He X. Rationale for Targeting the Wnt Signalling Modulator Dickkopf-1 for Oncology. Br J Pharmacol (2017) 174:4637–50. doi: 10.1111/bph.13894 PMC5727329 28574171

[17] Kitazawa K Nadanaka S Kadomatsu K Kitagawa H. Chondroitin 6-Sulfate Represses Keratinocyte Proliferation in Mouse Skin, Which is Associated With Psoriasis. Commun Biol (2021) 4:114. doi: 10.1038/s42003-020-01618-5 33495490 PMC7835381

[18] Nadanaka S Zhou S Kagiyama S Shoji N Sugahara K Sugihara K. EXTL2, a Member of the EXT Family of Tumor Suppressors, Controls Glycosaminoglycan Biosynthesis in a Xylose Kinase-Dependent Manner. J Biol Chem (2013) 288:9321–33. doi: 10.1074/jbc.M112.416909 PMC3611003 23395820

[19] Nadanaka S Hashiguchi T Kitagawa H. Aberrant Glycosaminoglycan Biosynthesis by Tumor Suppressor EXTL2 Deficiency Promotes Liver Inflammation and Tumorigenesis Through Toll-Like 4 Receptor Signaling. FASEB J (2020) 34:8385–401. doi: 10.1096/fj.201902076R 32347583

[20] Menck K Heinrichs S Baden C Bleckmann A. The WNT/ROR Pathway in Cancer: From Signaling to Therapeutic Intervention. Cells (2021) 10:142. doi: 10.3390/cells10010142 33445713 PMC7828172

[21] Oishi I Suzuki H Onishi N Takada R Kani S Ohkawara B. The Receptor Tyrosine Kinase Ror2 Is Involved in non-Canonical Wnt5a/JNK Signalling Pathway. Genes Cells (2003) 8:645–54. doi: 10.1046/j.1365-2443.2003.00662.x 12839624

[22] Pukrop T Klemm F Hagemann T Gradl D Schulz M Siemes S. Wnt 5a Signaling is Critical for Macrophage-Induced Invasion of Breast Cancer Cell Lines. Proc Natl Acad Sci USA (2006) 103:5454–9. doi: 10.1073/pnas.0509703103 PMC1459376 16569699

[23] Klemm F Bleckmann A Siam L Chuang HN Rietkotter E Behme D. Beta-Catenin-Independent WNT Signaling in Basal-Like Breast Cancer and Brain Metastasis. Carcinogenesis (2011) 32:434–42. doi: 10.1093/carcin/bgq269 21173432

[24] Comsa S Cimpean AM Raica M. The Story of MCF-7 Breast Cancer Cell Line: 40 Years of Experience in Research. Anticancer Res (2015) 35:3147–54. 26026074

[25] Hasan MK Widhopf GF 2nd Zhang S Lam SM Shen Z Briggs SP. Wnt5a Induces ROR1 to Recruit Cortactin to Promote Breast-Cancer Migration and Metastasis. NPJ Breast Cancer (2019) 5:35. doi: 10.1038/s41523-019-0131-9 31667337 PMC6814774

[26] Zhang S Chen L Cui B Chuang HY Yu J Wang-Rodriguez J. ROR1 is Expressed in Human Breast Cancer and Associated With Enhanced Tumor-Cell Growth. PloS One (2012) 7:e31127. doi: 10.1371/journal.pone.0031127 22403610 PMC3293865

[27] Fukuda T Chen L Endo T Tang L Lu D Castro JE. Antisera Induced by Infusions of Autologous Ad-CD154-Leukemia B Cells Identify ROR1 as an Oncofetal Antigen and Receptor for Wnt5a. Proc Natl Acad Sci U.S.A. (2008) 105:3047–52. doi: 10.1073/pnas.0712148105 PMC2268582 18287027

[28] Balakrishnan A Goodpaster T Randolph-Habecker J Hoffstrom BG Jalikis FG Koch LK. Analysis of ROR1 Protein Expression in Human Cancer and Normal Tissues. Clin Cancer Res (2017) 23:3061–71. doi: 10.1158/1078-0432.CCR-16-2083 PMC5440207 27852699

[29] Karvonen H Perttila R Niininen W Hautanen V Barker H Murumagi A. Wnt5a and ROR1 Activate Non-Canonical Wnt Signaling *via* RhoA in TCF3-PBX1 Acute Lymphoblastic Leukemia and Highlight New Treatment Strategies *via* Bcl-2 Co-Targeting. Oncogene (2019) 38:3288–300. doi: 10.1038/s41388-018-0670-9 30631148

[30] Sheikh MA Kong X Haymart B Kaatz S Krol G Kozlowski J. Comparison of Temporary Interruption With Continuation of Direct Oral Anticoagulants for Low Bleeding Risk Procedures. Thromb Res (2021) 203:27–32. doi: 10.1016/j.thromres.2021.04.006 33906063 PMC8225570

[31] Masiakowski P Yancopoulos GD. The Wnt Receptor CRD Domain Is Also Found in MuSK and Related Orphan Receptor Tyrosine Kinases. Curr Biol (1998) 8:R407. doi: 10.1016/S0960-9822(98)70263-5 9637909

[32] Saldanha J Singh J Mahadevan D. Identification of a Frizzled-Like Cysteine Rich Domain in the Extracellular Region of Developmental Receptor Tyrosine Kinases. Protein Sci (1998) 7:1632–5. doi: 10.1002/pro.5560070718 PMC2144063 9684897

[33] Theocharis AD Skandalis SS Tzanakakis GN Karamanos NK. Proteoglycans in Health and Disease: Novel Roles for Proteoglycans in Malignancy and Their Pharmacological Targeting. FEBS J (2010) 277:3904–23. doi: 10.1111/j.1742-4658.2010.07800.x 20840587

[34] Kastana P Choleva E Poimenidi E Karamanos N Sugahara K Papadimitriou E. Insight Into the Role of Chondroitin Sulfate E in Angiogenesis. FEBS J (2019) 286:2921–36. doi: 10.1111/febs.14830 30932321

[35] Vallen MJ Schmidt S Oosterhof A Bulten J Massuger LF Van Kuppevelt TH. Primary Ovarian Carcinomas and Abdominal Metastasis Contain 4,6-Disulfated Chondroitin Sulfate Rich Regions, Which Provide Adhesive Properties to Tumour Cells. PloS One (2014) 9:e111806. doi: 10.1371/journal.pone.0111806 25372710 PMC4221137

[36] Li J Sparkenbaugh EM Su G Zhang F Xu Y Xia K. Enzymatic Synthesis of Chondroitin Sulfate E to Attenuate Bacteria Lipopolysaccharide-Induced Organ Damage. ACS Cent Sci (2020) 6:1199–207. doi: 10.1021/acscentsci.0c00712 PMC7379384 32724854

[37] Weyers A Yang B Yoon DS Park JH Zhang F Lee KB. A Structural Analysis of Glycosaminoglycans From Lethal and Nonlethal Breast Cancer Tissues: Toward a Novel Class of Theragnostics for Personalized Medicine in Oncology? OMICS (2012) 16:79–89. doi: 10.1089/omi.2011.0102 22401653 PMC3300064

[38] Soares Da Costa D Reis RL Pashkuleva I. Sulfation of Glycosaminoglycans and Its Implications in Human Health and Disorders. Annu Rev BioMed Eng (2017) 19:1–26. doi: 10.1146/annurev-bioeng-071516-044610 28226217

[39] Mereiter S Balmana M Campos D Gomes J Reis CA. Glycosylation in the Era of Cancer-Targeted Therapy: Where Are We Heading? Cancer Cell (2019) 36:6–16. doi: 10.1016/j.ccell.2019.06.006 31287993

[40] Bafico A Liu G Yaniv A Gazit A Aaronson SA. Novel Mechanism of Wnt Signalling Inhibition Mediated by Dickkopf-1 Interaction With LRP6/Arrow. Nat Cell Biol (2001) 3:683–6. doi: 10.1038/35083081 11433302

[41] Kimura H Fumoto K Shojima K Nojima S Osugi Y Tomihara H. CKAP4 is a Dickkopf1 Receptor and Is Involved in Tumor Progression. J Clin Invest (2016) 126:2689–705. doi: 10.1172/JCI84658 PMC4922689 27322059

